# Is lactate an undervalued functional component of fermented food products?

**DOI:** 10.3389/fmicb.2015.00629

**Published:** 2015-06-19

**Authors:** Graciela L. Garrote, Analía G. Abraham, Martín Rumbo

**Affiliations:** ^1^Centro de Investigación y Desarrollo en Criotecnología de Alimentos (CIDCA - CONICET), Universidad Nacional de La PlataLa Plata, Argentina; ^2^Área Bioquímica y Control de Alimentos, Facultad de Ciencias Exactas, Universidad Nacional de La PlataLa Plata, Argentina; ^3^Instituto de Estudios Inmunológicos y Fisopatológicos (IIFP - CONICET), Universidad Nacional de La PlataLa Plata, Argentina

**Keywords:** fermentation, lactate, functional food, probiotics, bioactive properties

## Abstract

Although it has been traditionally regarded as an intermediate of carbon metabolism and major component of fermented dairy products contributing to organoleptic and antimicrobial properties of food, there is evidence gathered in recent years that lactate has bioactive properties that may be responsible of broader properties of functional foods. Lactate can regulate critical functions of several key players of the immune system such as macrophages and dendritic cells, being able to modulate inflammatory activation of epithelial cells as well. Intraluminal levels of lactate derived from fermentative metabolism of lactobacilli have been shown to modulate inflammatory environment in intestinal mucosa. The molecular mechanisms responsible to these functions, including histone deacetylase dependent-modulation of gene expression and signaling through G-protein coupled receptors have started to be described. Since lactate is a major fermentation product of several bacterial families with probiotic properties, we here propose that it may contribute to some of the properties attributed to these microorganisms and in a larger view, to the properties of food products fermented by lactic acid bacteria.

## Lactate is a Major Component of Lactic-Acid Bacteria Fermented Foods

For about 40 centuries, without understanding the scientific basis, people have been using lactic acid bacteria to produce fermented products that were originally developed as a way to preserve food from microbial or physicochemical modification that may alter its sensory or nutritional value. By fermentation, it has been possible to develop a wide variety of products of different taste, texture, and function. Lactic acid bacteria are traditionally used in obtaining dairy products from all over the world, including yogurt, cheese, butter, buttermilk, kefir, and koumiss, among others. Lactic acid bacteria refer to a large group of bacteria that share genetic traits and produce lactic acid as main end product of fermentation. They are widespread in nature and are also found in the gastrointestinal tract.

Fermenting milk with lactic acid bacteria provide a final product that contains lactic acid as a hallmark among other metabolites that may contribute to product characteristics. Although they are best known for their role in the preparation of fermented dairy products, lactic acid bacteria are also used in non-dairy food processing such as pickling of vegetables, curing fish, meats, and sausages as well as in traditional fermented products around the world such as pozole, pulque, chicha, gari, kimchi, among others (Nout, 2009). Furthermore, LAB is also a major contributor to fermentation process that takes place in ensilage (Jay et al., 2005). In the process of yogurt production, around 20% of lactose present in milk is transformed into lactic acid, and the content of lactic acid in yogurt is around 0.9% (Cheng, 2010). Other fermented milk product as kefir may reach to 2% of lactate (Garrote et al., 2010; Londero et al., 2012).

## Lactic-Acid Bacteria-Fermented Products have Beneficial Health Properties

The popular belief that fermented products have beneficial health effects is probably very old, but only in the past decades these ideas have begun to find a scientific support. During the last 20 years, a major expansion of food with health-promoting properties has taken place leading to the so-called “functional foods.” This type of food, consumed as part of normal daily diet, contains bioactive ingredients that offer health benefits.

Within functional foods, probiotics have acquired an important role, showing capacity to regulate metabolism and immunity of the consumer resulting in improvement of the quality of life (Tojo Sierra et al., 2003). Probiotics according to the World Health Organization (FAO/WHO Report, 2002) are live microorganisms which when administered in adequate doses confer beneficial effects on host health.

There have been proposed many mechanisms by which probiotics may contribute to consumer health, although for several of them the cellular and molecular bases are not completely elucidated. Probiotics may produce agents that suppress the growth of other microorganisms such as organic acid (Garrote et al., 2000) or other inhibitory compounds (Holzapfel et al., 1995; Beshkova and Frengova, 2012); furthermore, they can compete for receptors and binding sites with other intestinal microbes on the intestinal mucosa exerting a protective affect against pathogen infection (Golowczyc et al., 2007; Kakisu et al., 2013). Probiotics can modulate the intestinal immunity and alter the responsiveness of the intestinal epithelia and immune cells to microbes in the intestinal lumen (Thomas and Versalovic, 2010). In this regard, numerous studies have shown that lactic acid bacteria in fermented milk improve different parameters of immune function (Matar et al., 2001; Isolauri et al., 2004; Tsai et al., 2012).

The consumption of fermented food and/or probiotics also modifies the intestinal microbiota which plays an important role in the function and integrity of the gastrointestinal tract, maintenance of immune homeostasis and host energy metabolism (Hemarajata and Versalovic, 2013; Flint et al., 2015). Microbes in the gastrointestinal tract can exert numerous effects on different cells of the mucosal immune system and, in turn, induce the production of cytokines, which prime the innate immune response (O’Flaherty et al., 2010). Recent studies revealed that microbiota, including their metabolites, modulate key signaling pathways involved in the inflammation of the mucosa. The underlying molecular mechanisms of host–microbiota interactions are still not fully elucidated; however, manipulation of microbiota by probiotics or prebiotics is becoming increasingly recognized as an important therapeutic option, especially for the treatment of the dysfunction or inflammation of the intestinal tract (Kanauchi et al., 2013). The metabolic output of the modification of gut microbiota is the production of different profile of short chain fatty acids (SCFA) such as butyrate, propionate, and acetate. It has been reported that SCFA show anti-inflammatory properties (Maslowski and Mackay, 2011). Furthermore, it has been shown that metabolites present in the supernatants of fermented dairy products can exert a protective effect *ex vivo* on intestinal mucosa exposed to inflammatory insults (Tsilingiri et al., 2012). Based in these results, Rescigno and coworkers has recently proposed the concept of postbiotics, meaning metabolites produced upon microbial fermentation that may have bioactive capacity and that could be useful for modulation of host response in cases of inflammatory diseases (Tsilingiri and Rescigno, 2013).

Many factors can be involved in the health promoting properties of a fermented food, such as the presence of probiotic microorganisms themselves, the metabolites produced during fermentation, products coming from the hydrolysis of the components of the food matrix, or changes in the microbiota induced by any of these factors. Taking into account that lactate is the main metabolite of many fermented products, it is conceivable to ask if lactate plays a role in the health promoting properties of fermented food.

## Lactate has Bioactive Capacities Acting through Different Mechanisms

**FIGURE 1:**
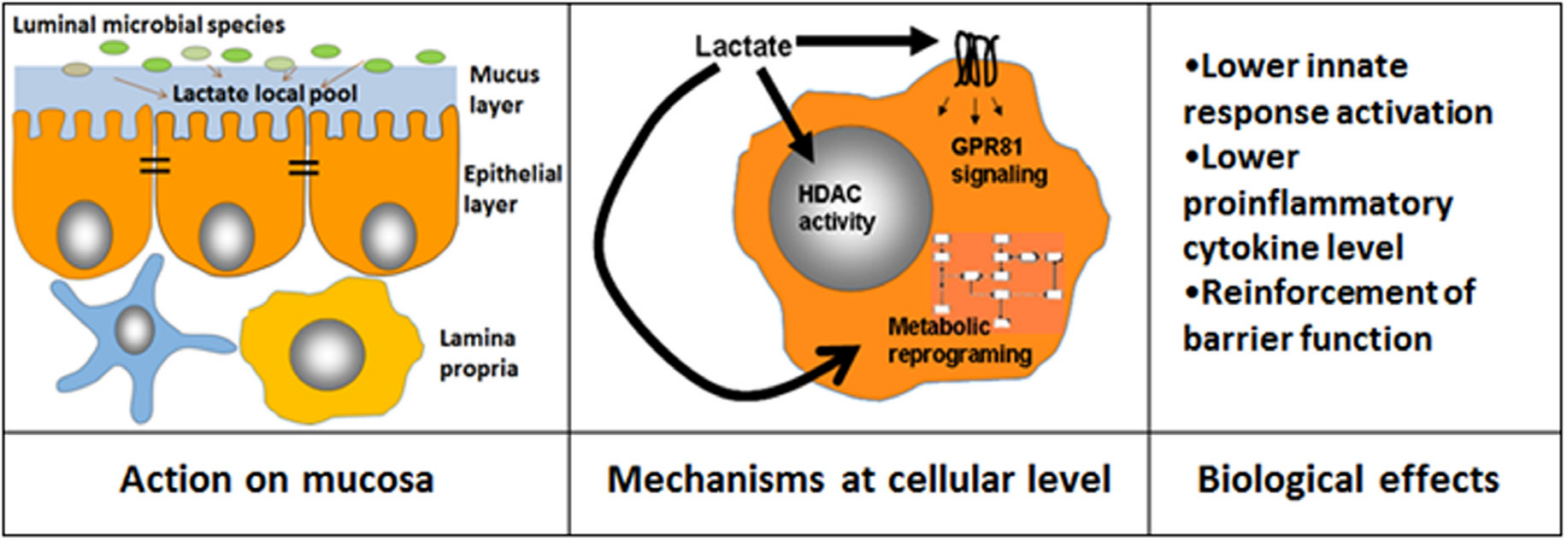
**Different mechanisms that mediate lactate bioactive effects**. Lactate luminal intestinal levels are contributed by lactate present in ingested food and also by that produced by intestinal microorganisms. The local lactate pool in the mucosal cellular environment is contributed by microbial species able to adhere to mucus/cell surface and may target epithelial cells as well as immune cells present in the lamina propria **(Left)**. Lactate may influence cellular activities by at least three independent ways: (i) by modulating gene expression through modification of histone deacetylase activity (HDAC), (ii) by triggering different signaling pathways by GPR81, (iii) by inducing changes in metabolic pathways such as reducing glycolysis rate **(Center)**. As a consequence of these cellular processes, different functional effects are achieved **(Right)**.

Lactate has been considered as a mere carbon metabolite with specific organoleptic/antimicrobial properties; however, different bioactive capacities of lactate have been recently shown (**Figure 1**). The lactic acid produced by the probiotic lactobacilli has been shown to be critical in modulating inflammation in a model of small intestine injury caused by indomethacin (Watanabe et al., 2009). We have recently shown that lactate abrogates TLR and IL1b dependent activation of intestinal epithelial cells (Iraporda et al., 2014). Moreover, besides immunomodulation, Okada et al. (2013) showed that luminal lactate stimulated enterocyte proliferation in a murine model of hunger-feedback, contributing to maintain intestinal barrier function. Beyond intestinal epithelial cells, lactate could have bioactive effects on myeloid cells. Lactate in the 10–20 mM range has been shown to modulate LPS-dependent monocyte activation (Dietl et al., 2010), whereas this activity is enhanced at pH 6.6 (Peter et al., 2015). In this case inhibition of NF-κB activation was also evidenced. Watanabe et al. (2009) also showed that lactate can modulate NF-κB signaling in myeloid cells. Furthermore, modulation of DC activation by lactate has also been described (Gottfried et al., 2006; Nasi et al., 2010; Nasi and Rethi, 2013; Iraporda et al., 2015).

Although there is evidence that lactate modulates key functions of main players of innate response such as myeloid and epithelial cells, the mechanisms responsible for these activities are still not yet fully elucidated but several options are possible (**Figure 1**). In recent years, several G protein-coupled receptors (GPCRs) have been characterized as sensors of small molecules such as fatty acids, sugars, or endogenous intermediate metabolites from microbial or food sources, having a profound impact on various biological processes (Blad et al., 2012). Among these receptors, GPR81 (or HCA1 or HCAR1) is specific for lactate (Offermanns, 2013), constituting an interesting candidate to mediate lactate bioactive effects. GPR81 is expressed primarily in adipocytes and have an antilipolytic effect (Liu et al., 2009). However, it has been shown that this receptor is also expressed in intestinal tissue (Iraporda et al., 2014) and it mediates macrophage dependent anti-inflammatory effects in mouse models of hepatitis and pancreatitis (Hoque et al., 2014). GPR81 dependent anti-inflammatory effects of lactate on macrophages are independent on G_i_ proteins and dependent on β-arrestin2 mediated signaling (Hoque et al., 2014; Liu et al., 2014). It still has to be confirmed if GPR81 may contribute to lactate bioactive properties observed in intestinal models of inflammation.

Beyond the signaling capacity through GPR81, lactate can also modulate histone deacetylase activity, showing specific patterns of gene expression regulation (Latham et al., 2012). Several modulatory effects on macrophages and epithelial cells were also associated with histone deacetylase capacity (Latham et al., 2012; Schilderink et al., 2013; Chang et al., 2014). Furthermore, high concentrations of lactate in extracellular milieu have also effects on modulation of cell metabolism, specially affecting glycolysis rate, which has been correlated with modulation of the production of proinflammatory mediators, such as TNFα by macrophages (Dietl et al., 2010). It has been recently shown that MCT4, a lactate membrane transporter, is induced in macrophages upon TLR activation and is critical for the management of lactate produced upon cell activation (Tan et al., 2015). Blocking the capacity of the macrophages to export lactate (Tan et al., 2015) or high concentrations of lactate in extracellular milieu (Dietl et al., 2010) have similar effects on cell metabolism that can contribute to modulation of proinflammatory mediator production. Furthermore, at systemic level lactate has other important regulatory actions on energy metabolism (Sola-Penna, 2008) that could also be triggered by enhanced intestinal lactate absorption. Changes in systemic levels of SCFA were reported by enhanced intestinal production and absorption (Macia et al., 2015), and this also could be the case for lactate.

## Considering Probiotic Properties from a Different Perspective

Taking into consideration the different bioactive properties of lactate mentioned above, a novel framework to interpret evidence on probiotic activity could be considered. So far, viability has been a major characteristic that has been the quintessence of probiotic action. Metabolic capacity, including fermentation is always dependent on microbial viability. As shown by Watanabe et al. (2009) and Flint et al. (2015), lactic acid production *in situ* is a key aspect for intestinal inflammation modulation by lactobacilli. Capacity to generate terminal fermentation metabolites has been shown to be critical for *in vivo* action of other microbial species with probiotic properties such as *Bifidobacterium* (Fukuda et al., 2011). Furthermore, adhesion to the intestinal mucosa is a desirable property for probiotic microorganisms and has been related to many of their health benefits (Servin and Coconnier, 2003). Autoaggregation and surface hydrophobicity of probiotic strains have been also correlated to adhesive capacity and have been also considered as positive traits in potentially probiotic strain selection studies (Kos et al., 2003; Collado et al., 2008; Botta et al., 2014; Papadimitriou et al., 2015). While it has been shown that adhesion to mucus/epithelial surface can be beneficial by blocking adhesion sites for potentially pathogens (Candela et al., 2008), the mechanistic basis of the immunomodulatory capacity associated to adhesion has been elusive. Without excluding other possible mechanisms that mediate this action, it is reasonable to assume that adhesion to epithelium increases the exposition of epithelial cells and intraepithelial leukocytes to bacterial fermentation products. Consequently, although luminal concentration of lactate may be high enough -at least in colon- to exert a modulatory activity on mucosa, the presence of adhesive bacteria with high metabolic capacity to produce lactate may even increase local concentration of this metabolite and consequently enhance immunomodulation, even with modest changes in luminal lactate concentration (Watanabe et al., 2009; Flint et al., 2015). Taking this into consideration, the evaluation of the capacity to use lactic acid fermentative pathway with high rate may be also considered as a desirable feature for probiotic candidate selection.

Beyond the capacity of probiotics to conduce lactic fermentation *in situ*, the health promoting effects of consumption of food containing relatively high amount of lactate such as yogurt, may be of considerable interest. In this case, the proximal mucosal sites, such as stomach will be exposed to highest concentration of lactate. In this case, the acid pH may also potentiate lactic acid modulatory effects (Dietl et al., 2010; Peter et al., 2015). Benefits of consumption of yogurt in different gastric inflammatory models have been reported, without establishing the mechanisms beyond this action (Uchida and Kurakazu, 2004; Uchida et al., 2010). On the other hand, lactate-rich food consumption may also impact in the microbiota composition independently of incorporation of viable bacteria, as has been shown by Garcia-Albiach et al. (2008) who compared gene structure of intestinal microbiota in groups receiving yogurt or heat-treated yogurt, finding comparable effects in both groups. Although in these cases many components of ingested food may contribute to microbiota shift, the presence of lactate, which is also an energy source for several intestinal microbial populations would possibly play a role.

So far, lactate bioactive properties have been disregarded. In the light of the accumulated evidence on lactate bioactivity and its mechanistic basis, it may be reasonable to reconsider the attribution of different properties of functional foods to different food components. Lactate as food component itself or as a bioactive metabolite generated *in situ* on the intestinal mucosa may contribute to health promoting-properties and should be valorised.

## Conflict of Interest Statement

The authors declare that the research was conducted in the absence of any commercial or financial relationships that could be construed as a potential conflict of interest.
